# Association between Maternal Exposure to Chemicals during Pregnancy and the Risk of Foetal Death: The Japan Environment and Children’s Study

**DOI:** 10.3390/ijerph182211748

**Published:** 2021-11-09

**Authors:** Tadao Ooka, Sayaka Horiuchi, Ryoji Shinohara, Reiji Kojima, Yuka Akiyama, Kunio Miyake, Sanae Otawa, Hiroshi Yokomichi, Zentaro Yamagata, on behalf of the Japan Environment and Children’s Study Group

**Affiliations:** 1 Department of Health Sciences, School of Medicine, University of Yamanashi, Chuo-shi 409-3898, Yamanashi, Japan; kojimar@yamanashi.ac.jp (R.K.); yukaa@yamanashi.ac.jp (Y.A.); kmiyake@yamanashi.ac.jp (K.M.); hyokomichi@yamanashi.ac.jp (H.Y.); zenymgt@yamanashi.ac.jp (Z.Y.); 2 Center for Birth Cohort Studies, University of Yamanashi, Chuo-shi 409-3898, Yamanashi, Japan; hsayaka@yamanashi.ac.jp (S.H.); rshinohara@yamanashi.ac.jp (R.S.); osanae@yamanashi.ac.jp (S.O.)

**Keywords:** foetal death, hair dye, stillbirth, spontaneous abortion, pregnancy, maternal exposure, chemical exposure, hairdressers

## Abstract

Scarce knowledge is available on the relationship between maternal chemical exposure during pregnancy and foetal deaths. We studied the association of spontaneous abortions and stillbirths with occupational or daily maternal exposure to chemicals commonly used by pregnant women. Data from the Japan Environment and Children’s Study (JECS), a nationwide prospective birth cohort study, were used. The participants of the study were asked about the frequency of their use of gasoline, pesticides, hair dye, and chlorine bleach during the first and the second to third trimesters of pregnancy. We investigated the relationship between the frequency of the use of chemicals and foetal death. Of the 104,065 foetuses, 923 (0.91%) were spontaneous abortions and 379 (0.37%) were stillbirths. Any type of exposure during the first trimester was not significantly associated with spontaneous abortions. Nevertheless, a more than weekly occupational use of hair dye from the first to the second/third trimester was significantly associated with stillbirth. The results of this study suggest that the frequent use of hair dye during pregnancy can have severe adverse effects on the foetus. These findings can help pregnant women, especially hairdressers, refrain from the continuous use of hair dyes.

## 1. Introduction

Foetal deaths, including spontaneous abortion and stillbirth, are considered to be among the most common and severe complications of pregnancy. Foetal deaths have significant economic and psychosocial effects on parents, families, healthcare providers, and the society as a whole [1]. The incidence of spontaneous abortion is 12–15% annually [2], and stillbirth occurs in 1.8% of all recognised pregnancies annually, indicating that approximately 2.6 million stillbirths occur worldwide every year [3]. The Every Newborn Action Plan has set a target of 12 or fewer stillbirths per 1000 births in every country by 2030 [4]; however, this is yet to be achieved. One of the major reasons for this is the knowledge gap on the causes and predictors of foetal death [5]. Previous studies have shown that maternal complications, such as age, obesity, hypertension, and diabetes, as well as maternal habits such as smoking are attributed to spontaneous abortion [2] and stillbirths [6,7].

Chemical exposures are also known to be attributed to foetal death. N-methyl-2-pyrrolidone included in petroleum refining and pesticide formulation has been reported to have acute toxicity and fetotoxicity [8]. Aniline derivatives included in hair dye and disinfectant by-products included in chlorine bleach also increase the risk of stillbirth [9]. On the other hand, a few previous cohort studies have examined the association between maternal chemical exposure during pregnancy and foetal deaths. Studies in the US [10] and Canada [11] have suggested an association between pesticides and stillbirth, and a study in China [12] suggested an association between petrochemicals and spontaneous abortion. A meta-analysis [13] of hairdressers with reproductive disorders also suggests that this occupation is significantly associated with the occurrence of foetal death throughout the gestation period. Nonetheless, these studies were limited in demonstrating a robust association because of the small sample size and the inability to appropriately adjust for confounding factors.

Pregnant women are often exposed to a variety of chemicals such as gasoline, pesticides, hair dye, and chlorine bleach. In Japan, these chemicals are commercially restricted or periodically measured in the manufacturing process. However, there are no surveillance systems in sales or service locations such as gas station, farm, beauty salon, and industrial laundry. There has been a recent increase in women’s participation in social activities, especially in these sales or service occupations. Therefore, many women can be exposed to harmful chemicals during pregnancy.

Owing to the difficulty of collecting adequate data on foetal deaths, a comprehensive survey has not been conducted on the chemicals that are frequently used in women’s daily lives or occupations. Therefore, a large-scale nationwide study is needed to clarify the impact of frequent exposure of chemicals on foetal death. In this study, we used the dataset of a large nationwide birth cohort in Japan to investigate the relationship between maternal exposure to common chemicals during pregnancy and the occurrence of spontaneous abortions and stillbirths. The overall study objective was to demonstrate an association between foetal death and these chemical exposures using data from a nationally representative cohort study.

## 2. Materials and Methods

### 2.1. Study Population

The database was derived from the Japan Environment and Children’s Study (JECS), a large-scale, prospective birth cohort study [14]. JECS was launched in January 2011 under the initiative of the JECS Working Group to investigate the effects of environmental factors on a child’s health and development. Fifteen Regional Centres across the nation participated in the study, and pregnant women who resided in the catchment areas of each centre were recruited and followed by the centres (Figure S1).

The initial assessments and those after a year since the study’s inception revealed the representativeness of the study population to the general population of Japan [15,16]. This study was registered in the UMIN Clinical Trials Registry (UMIN000030786). More detailed information including the JECS study design and study protocol are described elsewhere [14,15,16,17]. The JECS protocol was approved by the Institutional Review Board on Epidemiological Studies of the Ministry of the Environment, Japan, and by the ethics review boards of all participating institutions. Written informed consent was obtained from all the participants of the study. Between January 2011 and March 2014, we contacted pregnant women via cooperating health care providers and/or local government offices issuing Maternal and Child Health Handbooks and registered those willing to participate in our study.

Finally, data on 104,065 foetuses from 97,415 mothers were registered in JECS, which included 20,899 foetuses recruited in 2011, 31,225 foetuses in 2012, 36,242 foetuses in 2013, and 12,352 foetuses in 2014 (plus 3347 foetuses which had no data for the registered period). Foetuses with no confirmed data on delivery by the physician were excluded from this study. Although some pregnant women had multiple births or multiple deliveries during the same study period, we also included the data of those foetuses in the analyses because excluding these data would lead to a significant reduction in the number of foetuses at high risk. In the analysis, we set different study populations to study spontaneous abortion and stillbirth because the questionnaires about exposures corresponding to each outcome were distributed during different periods; furthermore, the target populations of the analyses differed based on each outcome. The study population for the outcome of spontaneous abortion excluded those cases with artificial abortion, while that for stillbirth excluded those with artificial abortion or spontaneous abortion.

### 2.2. Pregnancy Outcomes

Spontaneous abortion was defined as non-artificial foetal death before 22 weeks of gestation, and stillbirth was defined as foetal death on or after 22 weeks of gestation. The World Health Organization (WHO) recommends a definition of foetal death after 22 weeks of gestation for general statistics and for the registration of stillbirths [18]. The Japanese demographics and this study cohort also used the same definition. All births, including abortions and stillbirths, were confirmed by a physician, and data about the foetuses were obtained by the physician who examined them.

### 2.3. Maternal Exposure to Chemicals

In the study cohort, all pregnant women were asked to complete the personal details section in the questionnaire twice in the first trimester (at the entry to the JECS: 12–16 weeks of gestation) and during the second to third trimester (22–28 weeks of gestation) [19]. In each trimester, they were asked about the frequency of using or handling the following four kinds of substances in their occupation for more than half of a day since becoming pregnant: kerosene, petroleum, benzene, or gasoline; herbicides or insecticides; hair dye; and chlorine bleach or germicides. During the second to the third trimester, they were asked about the frequency of using or handling the following three kinds of substances daily: gasoline, herbicides or insecticides, and hair dye (at home or a beauty salon). Biomonitoring was not conducted in this cohort study because we asked women for each exposure using the questionnaire.

The options for the frequency of use in their occupations were ‘Never’; ‘1–3 times a month’; ‘1–6 times a week’; and ‘Every day’. Considering their frequency in terms of daily use, there were more detailed options for gasoline, herbicides, and insecticides. However, in this analysis, they were adjusted to the same frequency as used in the participants’ occupations. Regarding the daily exposure to hair dye, we asked about the exposure frequency both at home and at a beauty salon, with the options of ‘Never’, ‘Rarely’, ‘Sometimes’, and ‘Quite often’. A detailed description of the questions and options for chemical exposure is provided in the Supplementary Materials (Table S1).

### 2.4. Covariates

The adjustment factors were determined based on previous studies on spontaneous abortions [2] and stillbirths [6,7]. With regard to spontaneous abortion, the following adjustment factors were selected: maternal age and body mass index (BMI) prior to pregnancy, pre-existing hypertension and type 2 diabetes, smoking behaviour during pregnancy, weekly working hours, daily sitting hours (not including time spent asleep), maternal mental condition during pregnancy, parities, and multiple births. For stillbirths, in addition to these factors, the household income and educational background of the parents were also included as adjustment factors. These social factors were only included during the second/third trimester questionnaire since the questionnaire in the first trimester included the minimum necessary questions.

We used the K-6 Distress Scale [20] to determine participants’ mental condition. This scale assessed the mental status with a range of 0–24 points, with higher scores indicating a severe mental condition. The answers in the first trimester questionnaire on smoking behaviours, working hours, sitting hours, and mental conditions were used for the analyses of spontaneous abortions. On the other hand, the answers during the second/third trimester questionnaire were used for the analyses of stillbirth to adjust these covariates with the latest information. The adjustment factors of age, parity, BMI, K6 score, working hours, and sitting hours were treated as continuous variables, while the adjustment factors of multiple births and social factors were treated as categorical variables, as shown in Table 1. All subjects with one or more missing values in the adjustment variables were excluded from each analysis.

### 2.5. Statistical Analyses

The proportion of spontaneous abortions and stillbirths on each maternal characteristic and the relative risks of each category characteristic of spontaneous abortion and stillbirths were calculated. Moreover, the proportion of the frequency of chemical exposure in their occupations and their daily situation was also calculated.

For spontaneous abortions, univariate and multivariate logistic regression analyses were performed for each frequency of the occupational chemical exposure during the first trimester. A Cochran–Armitage trend test was also performed to determine the presence of a dose–response relationship by transforming each exposure category into a continuous variable (Table S1). Because of the extremely low frequency of the ‘Everyday’ option, it was merged with the ‘1–6 times a week’ option in the spontaneous abortion analyses.

For stillbirths, we conducted univariate and multivariate logistic regressions using each frequency of the occupational chemical exposure from the first to the second/third trimester. Since the ‘Everyday’ exposure frequency for the ‘Kerosene, Petroleum, Benzene, or Gasoline’ option was extremely low, it was merged with the ‘1–6 times a week’ option as ‘once a week and over’. In addition, univariate and multivariate logistic regressions were also conducted for daily exposure. The adjustment variables in the daily situation analyses were the same as those used in the analysis of occupational exposure. We also performed a trend test as in the case of spontaneous abortions. Furthermore, to consider the effect of missing answers, we performed a sensitivity analysis for occupational exposures with stillbirth by compensating for the missing answers for exposures during the second/third trimester with answers to the same questions in the first trimester.

As an additional analysis to examine the relationship between occupation and foetal death, we examined the association between the maternal occupation of agriculture or hairdressing and the occurrence of spontaneous abortions and stillbirths. The adjustment factors for these analyses were the same as those used in the spontaneous abortions or stillbirth analyses. The present study was based on the dataset of jecs-an-20180131, which was released in March 2018. For all analyses, the significance level was set at 5%. We used the Wald test to calculate *p*-values for all regression analyses. All analyses were performed using the R version 3.6.1 statistical package (R Foundation for Statistical Computing, Vienna, Austria.).

## 3. Results

Of the 104,065 foetuses registered in this study, 2285 foetuses were excluded because there was no information about the birth; a further 334 foetuses were excluded because they were artificially aborted. Consequently, 101,446 foetuses were eligible for descriptive statistics analysis (Figure S2).

Table 1 lists the incidence rate and relative risk of spontaneous abortions and stillbirths for each categorised risk factor. Of the 101,446 foetuses, 923 (0.91%) were spontaneous abortions and 379 (0.37%) were stillbirths. The relative risk of spontaneous abortions and stillbirths increased when pregnant women were over 30 years old. Parities were also associated with the occurrence of spontaneous abortion. However, a history of up to two parities was not associated with the occurrence of stillbirths. Multiple births increased the risk of both spontaneous abortions and stillbirths. A BMI of 30 or higher increased the risk of spontaneous abortion, and a BMI of 25 or higher increased the risk of stillbirth. A history of spontaneous abortions was also associated with both spontaneous abortions and stillbirths. Moreover, we noted an association between stillbirth and pre-existing type 2 diabetes and pregnancy-induced hypertension. We also noted an association between spontaneous abortions and sitting for 2–4 h daily during the first trimester. Alternatively, high K6 scores during the first trimester conferred a protective association for spontaneous abortion. Sitting for less than 2 h daily also showed a protective association for stillbirth.

The analyses of the spontaneous abortions were performed on 99,559 foetuses, with 422 spontaneous abortions noted after excluding 1887 foetuses with no information on the first trimester questionnaire. Any kind of chemical exposure during the first trimester did not significantly increase the risk of spontaneous abortions, in both the univariate and multivariate analyses (Table 2).

The analyses of stillbirths were performed on 98,499 foetuses, with 170 stillbirths noted after excluding 923 spontaneous abortions and 2024 foetuses with no information on the second/third trimester questionnaire. The use of hair dye in the participant’s occupations 1–6 times a week or every day from the first to the second/third trimester was significantly associated with stillbirths in the univariate model (1–6 times a week odds ratio: 4.71 (95% confidence interval: 1.74–12.77) vs. never; everyday OR: 4.96 [1.57–15.64] vs. never). This relationship was also shown in the adjusted model, and the adjustment enhanced the association (1–6 times a week OR: 4.92 [1.53–15.78] vs. never; everyday OR: 7.59 [2.32–24.85] vs. never). Furthermore, in the dose–response analyses, the risk of stillbirths significantly increased as the frequency of exposure increased in both the univariate model (*p* for trend: 0.0018) and the adjusted model (*p* for trend: 0.0011). Any other exposure to occupational chemicals was not significantly associated with stillbirths, regardless of the type and frequency (Table 3). Sensitivity analyses examining the effect of the missing values of occupational exposure did not change the significance of the results (Table S2).

Chemical exposures on a daily basis did not have a significant association with stillbirth, regardless of their types and frequencies (Table 4). Nonetheless, the group that ‘quite often’ used hair dye at home in the hair salons had a higher, albeit non-significant, risk of stillbirths than the other groups after adjustment (OR: 1.98 (0.62–6.29) for home use; OR: 1.92 (0.88–4.22) for use in hair salons). As regards the relationship between occupation and foetal death, there was a significant association between hairdressing and stillbirths in the adjusted model (OR: 3.56 (1.54–8.24)) (Table 5).

## 4. Discussion

In this nationwide cohort study in Japan, we investigated the relationship between chemicals commonly used in occupational or daily situations and foetal deaths, including spontaneous abortions and stillbirths. There were no significant associations between exposure to gasoline, insecticides or herbicides, or chlorine bleach and foetal death. However, maternal exposure to hair dye in their occupation from the first to the second/third trimester was significantly associated with the occurrence of stillbirths, and we also demonstrated a significant dose–response relationship. These associations were still significant after adjustment for risk factors.

In 2016, a UK research group reviewed demographic, environmental, nutritional, and lifestyle factors related to stillbirth [3]. The study revealed that maternal age, pre-pregnancy diabetes, and smoking during pregnancy increased the risk of stillbirth. This was consistent with the results of our study. Another UK cohort study [7] has shown that parities, a high maternal BMI, and previous history of foetal death are associated with an increased risk of stillbirth. This is also consistent with the results of our study. Furthermore, our study also suggested that pregnancy-induced hypertension was associated with the occurrence of stillbirth.

Our study does not suggest a relationship between foetal death and chemical exposures other than for hair dye. Nevertheless, previous studies in the US [10] and Canada [11] have suggested an association between pesticides and stillbirth, and a previous study in China [12] suggested an association between petrochemicals and spontaneous abortion. In our study, the respondents with high-frequency exposure to pesticides or petrochemicals included pregnant women in various occupations. The diversity of exposures by occupation and the different working environments between countries may be responsible for the differences observed compared with previous studies.

A meta-analysis [13] of hairdressers with reproductive disorders found that this occupation was significantly associated with the occurrence of foetal death throughout the gestation period, although the results were inconsistent for foetal death before 28 weeks. However, the study design had some limitations, including recall bias and limited adjustment factors in each study. In contrast, our study showed an association between occupation as a hairdresser and stillbirths on and after 22 weeks using data from a nationwide prospective cohort study with adequate adjustments, including social factors.

Moreover, in the present study, we showed that occupational exposure to hair dye was significantly associated with stillbirths. The main ingredient in hair dyes is aniline derivatives (known as oxidative dyes) in addition to alkaline agents and hydrogen peroxide as secondary ingredients. Among these ingredients, aniline derivatives, such as paraphenylenediamine, have severe adverse effects on human health [21]. In this study, we could not confirm the type of hair dye used by the pregnant women. However, most Japanese beauty salons use hair dye with paraphenylenediamine, and rarely use natural hair dyes like henna. Therefore, the exposure to hair dye in this study may be regarded as exposure to aniline derivatives including paraphenylenediamine. Aniline derivatives are known to cause methemoglobinemia following oral ingestion, transdermal absorption, and inhalation [22]. Some previous studies have reported them as emerging household poisons [23,24]. There are case reports of methemoglobinemia caused by the inhalation of aniline derivatives in newborns [25], and a recent one showed that the inhalation of hair dye by a hairdresser led to severe methemoglobinemia [26].

Foetuses are more susceptible to methemoglobinemia because of the high prevalence of foetal haemoglobin that is easily converted into methaemoglobin. Moreover, they have less methaemoglobin reductase (the enzyme that reduces methaemoglobin to haemoglobin) [27]. A pregnant hairdresser’s transdermal absorption or inhalation of hair dye may induce the placental passage of the aniline derivatives, which may convert the foetal haemoglobin into methaemoglobin, thus causing severe chronic oxygen deprivation in the foetus and resulting in stillbirth with respiratory or organ failure. Some previous studies have also suggested that elevated maternal methaemoglobin levels due to environmental substances can lead to foetal methemoglobinemia via the placental passage [28,29].

We found no association between the exposure to hair dye during pregnancy and spontaneous abortions in this study. The reason for this is unclear; however, the production of foetal haemoglobin begins at approximately six weeks of gestation and reaches its highest prevalence at approximately 26 weeks [30]. Consequently, the difference in the prevalence of foetal haemoglobin by pregnancy periods may cause a discrepancy in the influence of exposure on stillbirth and spontaneous abortions. Further investigation is needed to reveal the mechanisms between the exposure to aniline derivatives in hair dye and the occurrence of foetal death.

This is the first epidemiological study examining the relationship between maternal exposure to chemicals and both spontaneous abortions and stillbirths. This study used data from a nationwide cohort study with several study participants and appropriate adjustment factors. It is also the first study to reveal a significant relationship between hair dye exposure and the occurrence of stillbirths, including a dose–response relationship.

This study had several limitations; first, approximately half of the pregnant women who had spontaneous abortions or stillbirths were unable to answer the questionnaire on exposure frequency. This happened because the period of occurrence of the spontaneous abortions and stillbirths coincided with their questionnaire-answering period. Considering the answering period of each questionnaire, the number of spontaneous abortions before 16 weeks and stillbirths at 22–28 weeks may have been underestimated. Pregnant women who experienced spontaneous abortions early during gestation might have different characteristics and effects due to the exposures. Therefore, we might have different results in the spontaneous abortion analysis if we included them. Nevertheless, for stillbirth analyses, the impact on the results would be limited considering the result of the sensitivity analyses, compensating for the missing responses during the second/third trimester. This allowed almost all stillbirths to be included in the stillbirth analyses, and there was no change in association.

Second, because this study was an observational study of one ethnic group, there is a possibility of residual confounding and selection bias. It is difficult to overcome race limitations; however, to deal with residual confounding, we adjusted for the risk factors identified in the previous studies, socioeconomic factors, and occupation-related factors that were not considered in previous studies. We also examined the dose–response relationship to support the proof of causation.

Third, the exposures examined in the present study were based on responses to the questionnaire, and no environmental measurements or maternal chemical levels in the blood were measured. Therefore, it is unclear which components in the exposure would cause stillbirth, and it could be caused by other chemicals that are often used with the target chemicals in the same working situation (e.g., perm products and hair dye). In addition, we could not consider environmental exposure from living near agricultural properties or industrial facilities. However, the purpose of the present study was not to experimentally investigate the effects of a single substance on the mother or foetus, but to investigate the effects of commonly used occupational products such as hair dyes and insecticides.

Fourth, the medium of exposure, including the transdermal absorption or inhalation of the chemicals by pregnant women, also remains uncertain. As regards exposure to hair dye, the fact that most hairdressers in Japan wear protective gloves when dyeing hair and the availability of studies on methemoglobinemia following hair dye inhalation indicate that the effects following inhalation may be more significant. To clarify these unclear questions, we need to identify the relationship between exposure to hair dye and the maternal levels of aniline derivatives in blood, in addition to conducting animal studies on placental transferability and foetal toxicity.

Currently, it is unclear whether it should be recommended that pregnant hairdressers refrain from the continuous use of hair dyes. However, the results of this study suggest that frequent exposure to hair dye can have severe adverse effects on the foetus. They may, thus, be encouraged to refrain from these exposures after conception, and health authorities or decision makers should have greater control over the use of these chemical substances.

## 5. Conclusions

We showed an association between maternal occupational exposure to hair dye over a period of more than one week during pregnancy and stillbirths. Frequent exposure of pregnant women to hair dye, through transdermal absorption or inhalation, may adversely affect foetal development. To substantiate these results, further studies on the relationship between hair dye exposure and levels of related chemicals in the blood are required, as is evidence on their foetal toxicity.

## Figures and Tables

**Table 1:** Characteristics of the study population and risk factors associated with spontaneous abortion and stillbirth.

		Spontaneous Abortion			Stillbirth		
Risk Factors	No. of All Foetuses ^†^	No. of Spontaneous Abortions	Rate/1000 Births	Relative Risk (95% CI)	No. of Stillbirths	Rate/1000 Births	Relative Risk (95% CI)
Total	n = 101,446 (100%)	n = 923 (0.91%)	9.1		n = 379 (0.37%)	3.7	
**Maternal characteristics**							
Age (years):	n = 98,923 (97.5%)	n = 856			n = 374		
<20	1140 ^††^	5	4.4	0.71 (0.29–1.72)	3	2.6	0.98 (0.31–3.10)
20–24	10,255	65	6.3	1.02 (0.77–1.36)	31	3.0	1.13 (0.74–1.71)
25–29	29,082	180	6.2	ref.	78	2.7	ref.
30–34	34,454	283	8.2	1.33 (1.10–1.60)	140	4.1	1.52 (1.15–2.00)
35–39	20,462	251	12.3	1.98 (1.64–2.40)	94	4.7	1.72 (1.28–2.33)
≥40	3530	72	20.4	3.30 (2.51–4.32)	28	8.1	3.00 (1.95–4.61)
Parity:	n = 98,975 (97.6%)	n = 865			n = 367		
0	39,937	285	7.1	ref.	156	3.9	ref.
1	38,544	355	9.2	1.29 (1.11–1.51)	123	3.2	0.82 (0.65–1.04)
2	16,052	166	10.3	1.45 (1.20–1.75)	60	3.8	0.96 (0.71–1.29)
≥3	4442	59	13.3	1.86 (1.41–2.46)	28	6.4	1.62 (1.09–2.42)
Multiple births:	n = 101,446 (100%)	n = 923			n = 379		
1	99,476	878	8.8	ref.	343	3.5	ref.
≥2	1970	45	22.8	2.59 (1.92–3.48)	36	18.7	5.38 (3.82–7.56)
Body mass index:	n = 100,856 (99.4%)	n = 832			n = 374		
<18.5	14,903	123	8.3	1.02 (0.84–1.23)	45	3.0	0.86 (0.63–1.18)
18.5–24.9	74,700	606	8.1	ref.	263	3.5	ref.
25–29.9	8582	78	9.1	1.12 (0.89–1.42)	45	5.3	1.49 (1.09–2.04)
≥30	2671	36	13.5	1.66 (1.19–2.32)	21	8.0	2.25 (1.44–3.50)
Spontaneous abortion history *:	n = 98,886 (97.5%)	n = 419			n = 332		
0	78,969	289	3.7	ref.	248	3.2	ref.
1	15,649	95	6.1	1.66 (1.32–2.09)	58	3.7	1.18 (0.89–1.57)
≥2	4298	35	8.1	2.23 (1.57–3.16)	18	5.4	1.93 (1.29–2.89)
Pre-existing hypertension *:	n = 99,559 (98.1%)	n = 422			n = 336		
No	99,081	418	4.2	ref.	334	3.4	ref.
Yes	478	4	8.4	1.98 (0.74–5.29)	2	4.2	1.25 (0.31–4.99)
Pre-existing type2 diabetes *:	n = 99,559 (98.1%)	n = 422			n = 336		
No	99,424	420	4.2	ref.	333	3.4	ref.
Yes	135	2	14.8	3.51 (0.88–13.93)	3	22.6	6.71 (2.18–20.63)
Pregnancy-induced hypertension **:	n = 100,523 (99.1%)				n = 379		
No	97,299				359	3.7	ref.
Yes	3224				20	6.2	1.68 (1.07–2.63)
Gestational diabetes **:	n = 100,523 (99.1%)				n = 379		
No	97,777				371	3.8	ref.
Yes	2746				8	2.9	0.77 (0.38–1.55)
Pregnancy-related factors *							
Smoking during pregnancy: (first trimester/second trimester)	n = 98,820 (97.4%)/97,669 (96.3%)	n = 415			n = 166		
No	94,068/93,205	389	3.9	ref.	160	1.8	ref.
Yes	4752/4464	26	5.3	1.32 (0.89–1.97)	6	1.4	0.78 (0.35–1.77)
Drinking during pregnancy: (first trimester/second trimester)	n = 99,107 (97.7%)/97,661 (96.3%)	n = 415			n = 167		
No	89,287/94,936	374	4.0	ref.	165	1.9	ref.
Yes	9820/2725	41	4.2	1.00 (0.72–1.38)	2	0.8	0.42 (0.10–1.70)
Mental health score (K6) (first trimester/second trimester)	n = 98,833 (97.4%)/98,208 (96.8%)	n = 422			n = 170		
0–4	67,271/69,812	304	4.3	ref.	121	1.8	ref.
5–8	20,187/18,344	89	4.1	0.98 (0.77–1.23)	31	1.8	0.98 (0.66–1.45)
9–12	7921/6838	22	2.4	0.61 (0.40–0.95)	10	1.5	0.84 (0.44–1.61)
13–24	3454/3214	7	2.1	0.45 (0.21–0.95)	8	2.6	1.44 (0.70–2.93)
Occupation-related factors *							
Working hours per week (first trimester/second trimester)	n = 99,559 (98.1%)/98,095 (96.7%)	n = 422			n = 170		
0	39,085/46,668	175	4.1	ref.	82	1.8	ref.
0–20	7672/7199	36	4.8	1.05 (0.73–1.50)	8	1.2	0.63 (0.31–1.31)
20–40	32,833/28,234	125	3.7	0.85 (0.68–1.07)	55	1.9	1.11 (0.55–2.33)
≥40	19,969/15,994	86	4.2	0.96 (0.74–1.24)	25	1.6	0.89 (0.55–1.43)
Hours spent sitting per day (first trimester/second trimester)	n = 95,498 (94.1%)/94,185 (92.8%)	n = 407			n = 155		
≤2	18,868/16,199	79	4.2	1.16 (0.87–1.55)	18	1.1	0.55 (0.33–0.92)
2–4	27,013/22,619	133	4.9	1.36 (1.06–1.76)	32	1.4	0.70 (0.46–1.06)
4–6	20,243/20,387	89	4.4	1.22 (0.92–1.61)	34	1.7	0.82 (0.55–1.24)
≥6	29,374/34,980	106	3.6	ref.	71	2.0	ref.
Social factors ***							
Household income	n = 91,444 (90.1%)				n = 156		
<JPY 2 million	5182				12	2.3	1.40 (0.75–2.63)
JPY 2–4 million	31,586				63	2.0	1.21 (0.83–1.75)
JPY 4–6 million	30,223				50	1.7	ref.
JPY 6–10 million	20,547				25	1.2	0.74 (0.46–1.19)
≥JPY 10 million	3906				6	1.5	0.93 (0.40–2.16)
Maternal final education	n = 97,921 (96.5%)				n = 168		
Secondary school	4742				12	2.5	1.56 (0.84–2.90)
High school	32,449				55	1.7	1.05 (0.73–1.51)
Vocational school	22,396				39	1.7	1.08 (0.72–1.61)
University and above	38,334				62	1.6	ref.
Paternal final education	n = 91,864 (90.6%)				n = 163		
Secondary school	7110				13	2.0	1.17 (0.64–2.14)
High school	37,926				70	2.0	1.17 (0.82–1.66)
Vocational school	17,780				25	1.5	0.88 (0.55–1.41)
University and above	34,483				55	1.7	ref.

* Approximately half of the pregnant women who had a stillbirth or spontaneous abortion were unable to complete the questionnaire because the foetal death happened during or before their answering period. ** We did not calculate for spontaneous abortion because the incidence of these diseases at less than 22 weeks of gestation is considerably less than the incidence after 22 weeks. *** These factors were only included during the second/third trimester questionnaire. **^†^** Parentheses indicate the proportion of foetuses for which there are data on each variable. **^††^** The minimum age of the participating mothers was 14 years, and the maximum was 50 years. CI, confidence interval.

**Table 2:** Adjusted odds ratio of spontaneous abortion occurrence for occupational exposure during the first trimester.

Type and Frequency of Exposure	No. of Spontaneous Abortions/Total No. of Pregnancies	Rate/1000 Births	Crude Model		Adjusted Model *	
			OR (95% CI)	*p* for Trend	OR (95% CI)	*p* for Trend
**Occupational exposure during the first trimester**						
Kerosene, petroleum, benzene, or gasoline	n = 365/89,196	4.1				
Never	325/80,156	4.1	ref.	0.51	ref.	0.37
1–3 times a month	30/7053	4.3	1.05 (0.72–1.53)		1.07 (0.72–1.61)	
Once a week and over	10/1987	5.0	1.24 (0.66–2.33)		1.37 (0.70–2.67)	
Herbicides or insecticides	n = 363/88,620	4.1				
Never	336/83,400	4.0	ref.	0.19	ref.	0.29
1–3 times a month	22/4385	5.0	1.25 (0.81–1.92)		1.24 (0.79–1.95)	
Once a week and over	5/835	6.0	1.49 (0.61–3.61)		1.32 (0.49–3.56)	
Hair dye	n = 364/88,986	4.1				
Never	343/82,781	4.1	ref.	0.49	ref.	0.73
1–3 times a month	17/5300	3.2	0.77 (0.47–1.26)		0.80 (0.48–1.35)	
Once a week and over	4/905	4.4	1.07 (0.4–2.87)		1.25 (0.46–3.40)	
Chlorine bleach or germicide	n = 371/89,448	4.1				
Never	298/72,732	4.1	ref.	0.65	ref.	0.96
1–3 times a month	51/11,659	4.4	1.07 (0.79–1.44)		1.06 (0.77–1.44)	
Once a week and over	22/5057	4.4	1.06 (0.69–1.64)		0.93 (0.57–1.52)	

* Adjusted for age, BMI, pre-pregnancy type 2 diabetes/hypertension, maternal smoking during the first trimester, working hours per week, sitting hours per day, K6, parity, and multiple births. OR, odds ratio; CI, confidence interval; BMI, body mass index.

**Table 3:** Adjusted odds ratios for the occurrence of stillbirths for occupational exposures from the first to the second/third trimester.

	No. of Stillbirths Total No. of Pregnancies	Rate/1000 Births	Crude Model		Adjusted Model *	
Type and Frequency of Exposure			OR (95% CI)	*p* for Trend	OR (95% CI)	*p* for Trend
**Occupational exposure from the first to the second/third trimester**						
Kerosene, petroleum, benzene, or gasoline	n = 161/94,548	1.7				
Never	131/78,569	1.7	ref.	0.53	ref.	0.95
1–3 times a month	23/12,532	1.8	1.1 (0.71–1.72)		1.07 (0.64–1.78)	
Once a week and over	7/3447	2.0	1.22 (0.57–2.61)		0.95 (0.35–2.60)	
Herbicides or insecticides	n = 160/93,266	1.7				
Never	143/84,902	1.7	ref.	0.53	ref.	0.24
1–3 times a month	15/7228	2.1	1.23 (0.72–2.1)		1.45 (0.83–2.53)	
Once a week and over	2/1136	1.8	1.05 (0.26–4.23)		1.26 (0.31–5.15)	
Hair dye	n = 161/92,899	1.7				
Never	138/83,639	1.6	ref.	0.0018	ref.	0.0011
1–3 times a month	16/8373	1.9	1.16 (0.69–1.94)		1.14 (0.62–2.08)	
1–6 times a week	4/518	7.7	4.71 (1.74–12.77)		4.92 (1.53–15.78)	
Everyday	3/369	8.1	4.96 (1.57–15.64)		7.59 (2.32–24.85)	
Chlorine bleach or germicide	n = 162/94,619	1.7				
Never	125/69,454	1.8	ref.	0.45	ref.	0.72
1–3 times a month	27/18,955	1.4	0.79 (0.52–1.2)		0.91 (0.58–1.41)	
1–6 times a week	8/5491	1.5	0.81 (0.4–1.65)		0.92 (0.43–2.00)	
Everyday	2/719	2.8	1.55 (0.38–6.27)		1.00 (0.14–7.20)	

* Adjusted for age, BMI, pre-pregnancy type 2 diabetes/hypertension, maternal smoking during the second trimester, working hours per week, sitting time per day, K6, parity, multiple births, household income, and educational background of parents. OR, odds ratio; CI, confidence interval; BMI, body mass index.

**Table 4:** Adjusted odds ratio of stillbirth occurrence for exposure to daily life from the first to the second/third trimester.

	No. of Stillbirths/Total No. of Pregnancies	Rate/1000 Births	Crude Model		Adjusted Model *	
Type and Frequency of Exposure			OR (95% CI)	*p* for Trend	OR (95% CI)	*p* for Trend
**Exposure in daily life from the first to the second/third trimester**						
Gasoline	n = 166/98,122	1.7				
Never	89/54,315	1.6	ref.	0.51	ref.	0.61
1–3 times a month	53/32,063	1.7	1.01 (0.72–1.42)		1.12 (0.77–1.63)	
Once a week and over	24/11,744	2.0	1.25 (0.79–1.96)		1.06 (0.60–1.88)	
Herbicides or insecticides	n = 162/97,660	1.7				
Never	165/95,829	1.7	ref.	0.52	ref.	0.36
once a month and over	2/1831	1.1	0.86 (0.54–1.37)		0.40 (0.06–2.84)	
Hair dye—for home use	n = 168/98,068	1.7				
Never	124/75,775	1.6	ref.	0.20	ref.	0.35
Rarely	16/9206	1.7	1.06 (0.63–1.79)		1.03 (0.56–1.89)	
Sometimes	25/11,840	2.1	1.29 (0.84–1.98)		1.16 (0.69–1.95)	
Quite often	3/1247	2.4	1.47 (0.47–4.63)		1.98 (0.62–6.29)	
Hair dye: for use in hair salons	n = 168/98,034	1.7				
Never	84/48,838	1.7	ref.	0.72	ref.	0.28
Rarely	28/17,169	1.6	0.95 (0.62–1.45)		1.11 (0.69–1.78)	
Sometimes	47/28,857	1.6	0.95 (0.66–1.35)		1.09 (0.73–1.65)	
Quite often	9/3170	2.8	1.65 (0.83–3.29)		1.92 (0.88–4.22)	

* Adjusted for age, BMI, pre-pregnancy type 2 diabetes/hypertension, maternal smoking during the second trimester, household income, educational background of parents, hours worked per week, sitting time per day, K6, number of births, and multiple births. OR, odds ratio; CI, confidence interval; BMI, body mass index.

**Table 5:** Association between the occurrence of spontaneous abortion and stillbirths and exposure-related occupations among pregnant women.

	No. of Spontaneous Abortions or Stillbirths/Total No. of Pregnancies	Rate/1000 Births	Crude Model	Adjusted Model *
Occupations of Pregnant Women			OR (95% CI)	OR (95% CI)
**Occurrence of spontaneous abortion**				
Agriculture				
Yes	2/397	5.0	1.3 (0.32–5.24)	1.18 (0.29–4.79)
No	249/64,160	3.9	ref.	ref.
Hairdresser				
Yes	4/1383	2.9	0.74 (0.27–1.99)	0.83 (0.31–2.23)
No	247/63,174	3.9	ref.	ref.
**Occurrence of stillbirth**				
Agriculture				
Yes	2/395	5.1	1.47 (0.36–5.95)	3.51 (0.85–14.5)
No	220/63,911	3.4	ref.	ref.
Hairdresser				
Yes	8/1379	5.8	1.71 (0.84–3.47)	3.56 (1.54–8.24)
No	214/62,927	3.4	ref.	ref.

* Spontaneous abortion analyses are adjusted for age, BMI, pre-pregnancy type 2 diabetes/hypertension, maternal smoking during the second trimester, working hours per week, sitting time per day, K6, parity, multiple births, household income, and educational background of parents. Stillbirth analyses are adjusted for age, BMI, pre-pregnancy hypertension, maternal smoking during the second trimester, working hours per week, sitting time per day, K6, parity, multiple births, household income, and educational background of parents. Pre-pregnancy diabetes could not be included as an adjusted variable in the stillbirth analyses because of its small number of cases. OR, odds ratio; CI, confidence interval; BMI, body mass index.

## Data Availability

Data are unsuitable for public deposition due to the ethical restrictions and legal framework of Japan. It is prohibited by the Act on the Protection of Personal Information (Act No. 57 of 30 May 2003, amendment on 9 September 2015) to publicly deposit data containing personal information. Ethical Guidelines for Medical and Health Research Involving Human Subjects enforced by the Japan Ministry of Education, Culture, Sports, Science and Technology, and the Ministry of Health, Labour and Welfare also restricts the open sharing of epidemiological data. All inquiries about access to data should be sent to: jecs-en@nies.go.jp. The person responsible for handling enquiries sent to this e-mail address is Shoji F. Nakayama, JECS Programme Office, National Institute for Environmental Studies.

## Supplementary Materials

The following are available online at https://www.mdpi.com/article/10.3390/ijerph182211748/s1, Figure S1: Geographical location of regional centres; Figure S2: Flowchart of study participant selection; Table S1: Summary of questionnaires related to chemical exposure; Table S2: Sensitivity analysis of adjusted odds ratios for stillbirth occurrences for occupational exposures from the first to the second/third trimester.
