# Supplementary material for: Association between Maternal Exposure to Chemicals during Pregnancy and the Risk of Foetal Death: The Japan Environment and Children’s Study
Source: Int J Environ Res Public Health. 2021 Nov 9;18(22):11748. doi: 10.3390/ijerph182211748 (PMC8618242; doi:10.3390/ijerph182211748)
Supplement: Supplementary file 1 [file ijerph-18-11748-s001.zip › ijerph-1410797-supplementary.pdf]

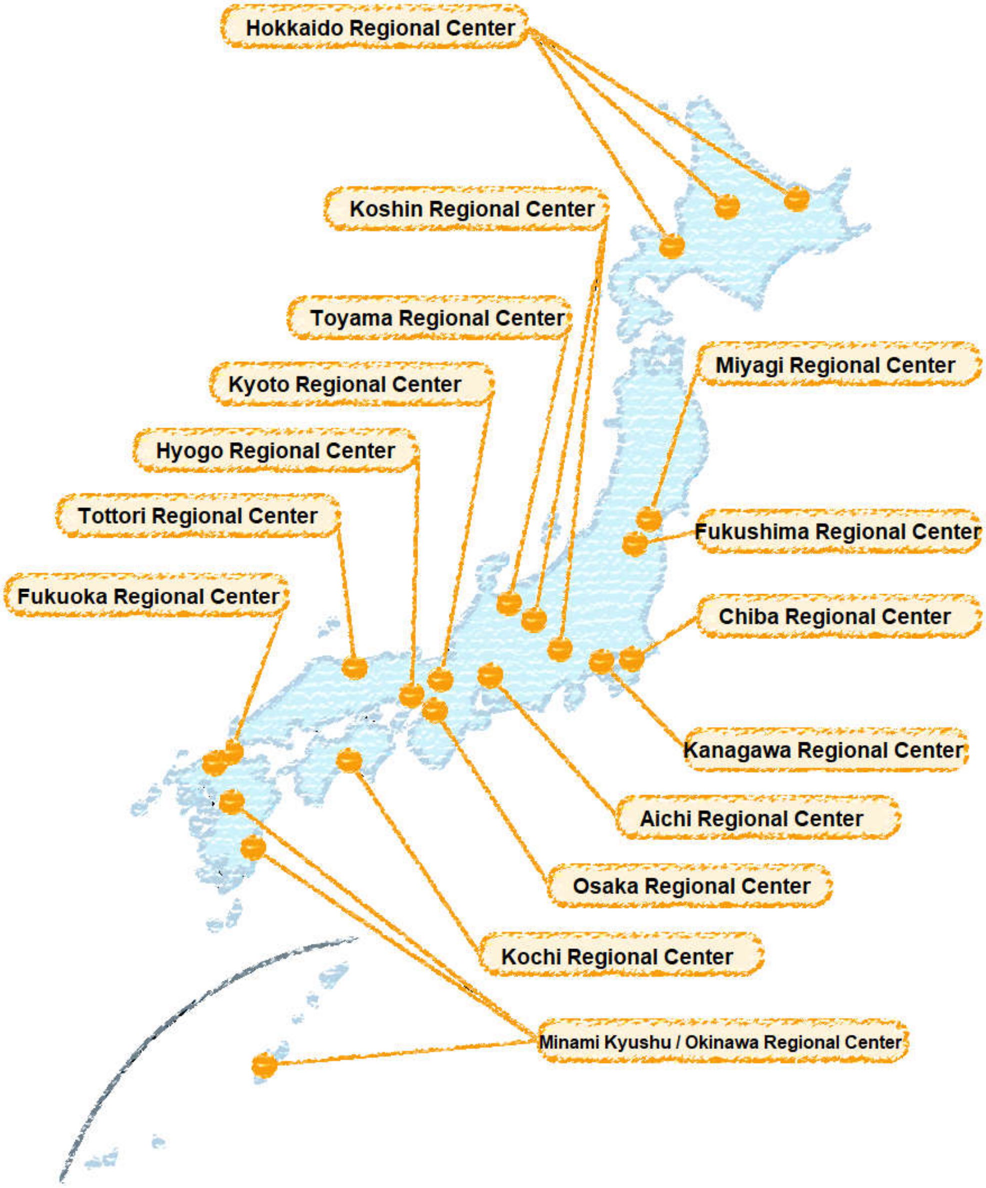

Figure S1. Geographic location of Regional Centres.

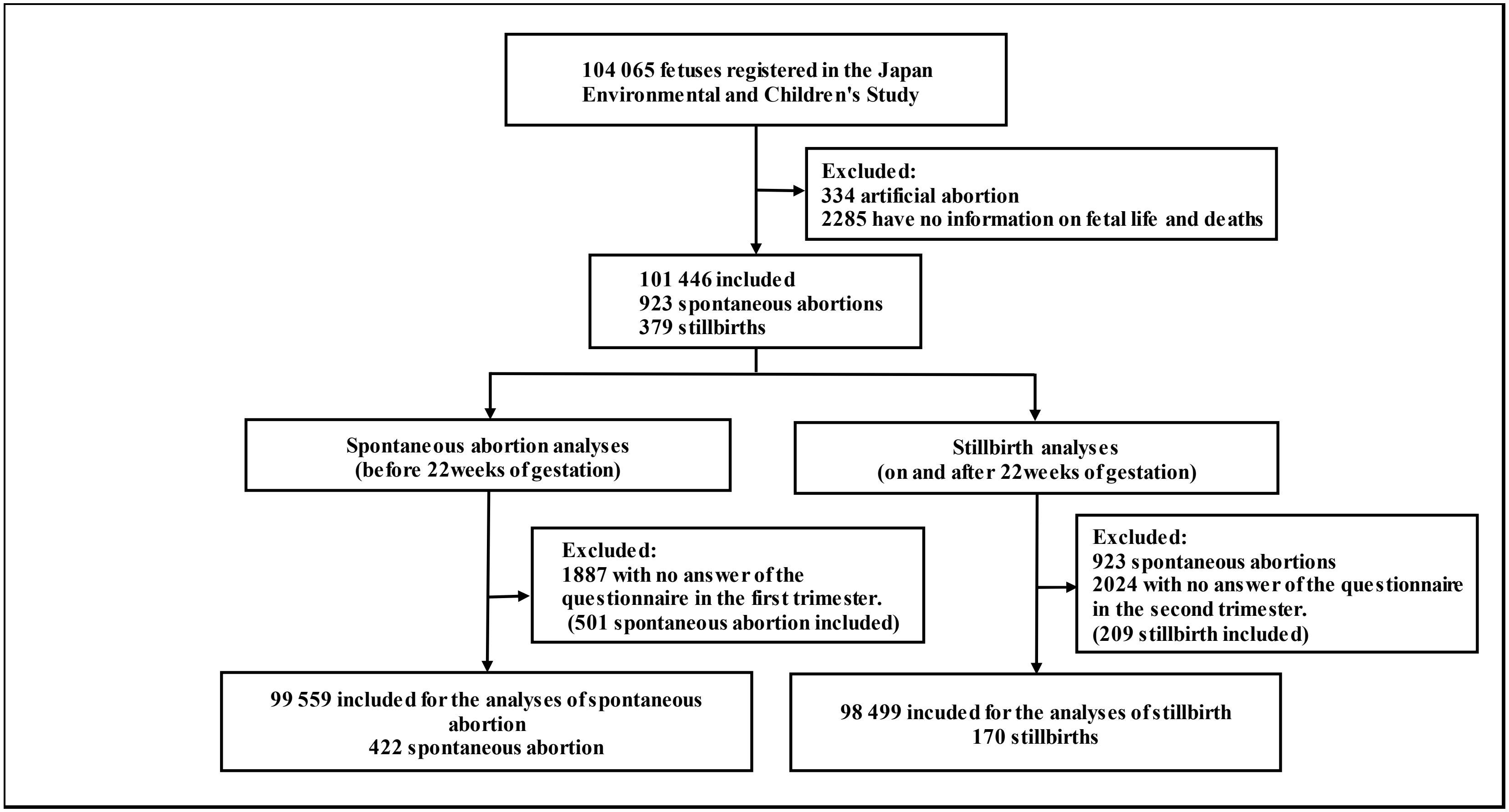

Figure S2. Flowchart of study participant selection.

Table S1. Summary of questionnaires related to chemical exposure.

Questionnaire in the first trimester (12-16 weeks of gestation)

Variable	Questions/Frequency options
**Occupational exposure**	Please select the frequency of using or handling the following materials during work for more than half a day since becoming pregnant,
Kerosene, Petroleum, Benzene, Gasoline	1 = Never, 2 = 1 to 3 times a month, 3 = 1 to 6 times a week, 4 = Everyday
Herbicides or Insecticides	1 = Never, 2 = 1 to 3 times a month, 3 = 1 to 6 times a week, 4 = Everyday
Hair dye	1 = Never, 2 = 1 to 3 times a month, 3 = 1 to 6 times a week, 4 = Everyday
Chlorine bleach, Germicide	1 = Never, 2 = 1 to 3 times a month, 3 = 1 to 6 times a week, 4 = Everyday

Questionnaire in the second/third trimester (22-28 weeks of gestation)

Variable	Questions/Frequency options
**Occupational exposure**	Please select the frequency of using or handling the following materials during work for more than half a day since becoming pregnant,
Kerosene, Petroleum, Benzene, Gasoline	1 = Never, 2 = 1 to 3 times a month, 3 = 1 to 6 times a week, 4 = Everyday
Herbicides or Insecticides	1 = Never, 2 = 1 to 3 times a month, 3 = 1 to 6 times a week, 4 = Everyday
Hair dye	1 = Never, 2 = 1 to 3 times a month, 3 = 1 to 6 times a week, 4 = Everyday
Chlorine bleach, Germicide	1 = Never, 2 = 1 to 3 times a month, 3 = 1 to 6 times a week, 4 = Everyday
**Exposure in daily life**	Please select the frequency of using or handling the following materials since becoming pregnant
	Refuelling a car with gasoline at a self-service gas station
Gasoline	1 = Everyday, 2 = 4-6 times a week, 3 = 2-3 times a week, 4 = Once a week, 5 = 1-3 times a month, 6 = Less than once a month, 7 = Never

Herbicides or Insecticides	Using a herbicide or a gardening pesticide in a garden, balcony, or farm<br>1 = Everyday, 2 = A few times a week, 3 = Once a week, 4 = 1-3 times a month,<br>5 = Less than once a month
Hair dye: for home use	Using of hair colouring products (hair dye) or perm solutions at home<br>1 = Quite often, 2 = Sometimes, 3 = Rarely, 4 = Never
Hair dye: for use in hair salons	Colouring or perming hair at a beauty salon<br>1 = Quite often, 2 = Sometimes, 3 = Rarely, 4 = Never

Table S2. Sensitivity analysis of adjusted odds ratios for stillbirth occurrences for occupational exposures from the first to the second/third trimester.

Type and frequency of exposure*	No. of stillbirths / total no. of pregnancies	Rate/1000 births	Crude model		Adjusted model**	
			OR (95% CI)	p for trend	OR (95% CI)	p for trend
**Occupational exposure from the first to the second/third trimester**						
Kerosene, Petroleum, Benzene, or Gasoline	n = 324/97 886	3·3				
Not at all	272/81 583	3·3	ref.		ref.	
1–3 times a month	40/12 777	3·1	0·93 (0·67–1·31)	0·85	1·05 (0·64–1·74)	0·99
once a week and over	12/3526	3·4	1·02 (0·57–1·82)		0·90 (0·33–2·46)	
Herbicides or Insecticides	n = 323/97 623	3·3				
Not at all	296/89 019	3·3	ref.		ref.	
1–3 times a month	25/7430	3·4	1·01 (0·67–1·52)	0·58	1·41 (0·81–2·46)	0·27
once a week and over	2/1174	1·7	0·51 (0·13–2·05)		1·23 (0·30–5·02)	
Hair dye	n = 325/97 626	3·3				
Not at all	285/87 965	3·2	ref.		ref.	
1–3 times a month	30/8716	3·4	1·06 (0·73–1·55)	0·0090	1·11 (0·61–2·02)	0·00034
1–6 times a week	6/554	10·8	3·37 (1·49–7·59)		6·44 (2·33–17·81)	
Everyday	4/391	10·2	3·18 (1·18–8·57)		7·47 (2·29–24·38)	
Chlorine bleach or Germicide	n=325/97 920	3·3				
Not at all	252/72 159	3·5	ref.	0·57	ref.	0·65
1–3 times a month	46/19 347	2·4	0·68 (0·5–0·93)		0·9 (0·58–1·39)	

1–6 times a week	24/5667	4·2	1·21 (0·8–1·85)	0·90 (0·42–1·94)
Everyday	3/747	4·0	1·15 (0·37–3·60)	0·97 (0·14–7·03)

*Compensating for deficiencies in the questionnaire of occupational exposure during the second/third trimester with answers to the same questionnaire in the first trimester. The response concordance rates of 'Kerosene, Petroleum, Benzene, or Gasoline', 'Herbicides or Insecticides', 'Hair dye', and 'Chlorine bleach or Germicide' between the first and second/third trimester's questionnaires for the same pregnant women are 72·7%, 89·0%, 89·3%, and 82·8%, respectively.

**Adjusted for age, BMI, pre-pregnancy type 2 diabetes/hypertension, smoking/drinking during the second trimester, working hours per week, sitting time per day, K6, parity, multiple births, household income, and educational background of parents.
